# Effects of Hydatid Cyst Fluid on Inflammation and Epithelial-Mesenchymal Transition in Colorectal Adenocarcinoma (Caco-2) Cell Line

**DOI:** 10.1007/s11686-025-01086-z

**Published:** 2025-07-07

**Authors:** Emin Yagmur, İpek Baysal, Serra Örsten, Fatma İnanç Tolun

**Affiliations:** 1 Institute of Health Sciences, Department of Medical Biochemistry, Kahramanmaraş Sutcu İmam University, Kahramanmaraş, Türkiye; 2 Vocational School of Health Services, Hacettepe University, Ankara, Türkiye; 3 Graduate School of Health Sciences, Department of One Health, Hacettepe University, Ankara, Türkiye; 4 Faculty of Medicine, Department of Medical Biochemistry, Kahramanmaras Sutcu İmam University, Kahramanmaras, Türkiye

**Keywords:** Colorectal cancer, EMT, Hydatid cyst fluid, Cytokine, Inflammation, *Echinococcus granulosus*

## Abstract

**Purpose:**

Anti-tumor immune responses and certain pathogens can exhibit various effects, including signals that reduce the risk of tumor formation or lead to cancer regression. Multiple studies have reported that infectious agents and the products of a wide range of host structures can modulate cancer development and growth positively or negatively and regulate the activation of immune responses. Numerous studies have reported that the parasite *Echinococcus granulosus* may have anti-cancer or carcinogenic effects on cancer cell proliferation in various cell cultures and animal models. The primary purpose of the study is to investigate the effect of animal-derived hydatid cyst fluid (HCF) at various concentrations (1/2, 1/3, 1/5) on the viability of human colorectal adenocarcinoma (Caco-2) and human colon epithelial (CoEpi) cell lines using the cell proliferation assay (XTT).

**Methods:**

Subsequently, the study aims to investigate cytokine concentrations and gene expression profiles of inflammatory cytokines TNF-α and IL-4, as well as epithelial-mesenchymal transition (EMT) regulatory signaling proteins TGF- β1, vimentin and E-cadherin, using ELISA and RT-PCR methods, respectively.

**Results:**

Following the HCF application, EMT was consistently detected in the Caco-2 cell line compared to the CoEpi cell line in the 1/5 volume application group, as confirmed by ELISA, RT-PCR and cell proliferation assays. On the other hand, a linear relationship was observed between the levels of TGF-β1 and TNF-α, which regulate the pro-inflammatory signaling mechanism based on the cell micro-environment and the decrease in cell viability. As the HCF concentration volume decreased (1/5), an increase in cell viability was observed (*P* < 0.01), along with an increase in TGF-β1 and TNF-α levels. Otherwise, in Caco-2 cells, as the HCF application concentrations increased (1/2), significant decreases in TGF-β1 and TNF-α levels, as well as in cell viability, were observed (*P* < 0.01).

**Conclusions:**

In this context, the common antigen-receptor structure between *E. granulosus* and cancer may modulate the signals of the immune response it regulates, affecting immune system cells and contributing to the progression of tumor cells.

## Introduction

Cystic Echinococcosis (CE) is a zoonotic infectious disease caused by the larval form of parasites belonging to the *Echinococcus granulosus sensu lato* (s.l.) species complex in intermediate hosts and humans [1]. *E. granulosus* is typically a common type of parasite found among domestic or wild animals. When the adult form of the *E. granulosus* parasite infects a host, it establishes itself in the host’s small intestine under suitable conditions and spreads its eggs into the circulation, thereby infesting the intermediate host. Except in extreme climatic conditions, the eggs can remain effective for a long time without degrading in the environment [2, 3]. During the infection process, the clear, antigen-rich hydatid cyst fluid (HCF) contained within hydatid cyst vesicles is produced by the metacestode stage of *E. granulosus* [3]. Common antigens found in *E. granulosus* and certain tumor types have been reported with various evidence indicating that they modulate immune responses and promote anti-cancer activity. However, according to the results obtained, it has also been reported that there may be carcinogenic effects in different in vivo or in vitro models studied [4]. It has also been reported in several studies that HCF modulates epithelial-mesenchymal transition (EMT) in the Caco-2 cell line [5]. Though, the mechanisms underlying this transformation have not been investigated regarding their role in inflammatory processes. Understanding the inflammatory processes involved in cancer development is important in this context. Cytokines are signaling proteins that mediate immunity, inflammation, and hematopoiesis, and they regulate various components of these processes. Inflammatory cytokines induce the initiation and progression of the inflammation mechanism within the cell by modulating various pathways and downstream signaling proteins [6]. EMT is a central ingredient of embryonic development, wound healing, and tumor cell migration. It is frequently noted in the literature that anti-inflammatory and pro-inflammatory cytokines have important antagonistic roles in regulating the development of EMT [7].

Additionally, several in vivo investigations have demonstrated that complete HCF or its 78-kDa fraction has anti-tumor effects in BALB/c-CT26 models [8, 9]. However, another study shows that lyophilized fertilized HCF increased human H1299 lung cancer cells’ migration, proliferation, and EMT-like alterations [10] Because of these reciprocal effects, the overall effect of HCF is still unknown and most likely context specific. Thus, going back to a strictly regulated in vitro platform becomes crucial. To close a mechanistic gap left by previous whole animal or single endpoint studies, we profile inflammatory cytokines and EMT markers in both malignant Caco-2 and non-malignant CoEpi colon cells.

The aim of the study involves examining the potential effects of animal-derived HCF application at various concentrations on the proliferation of human colorectal adenocarcinoma (Caco-2) and human colon epithelial (CoEpi) cell lines. Additionally, it is aimed to determine the activities of inflammatory cytokines TNF-α, IL-4, and the regulatory signaling proteins of the transition, TGF-β1, vimentin, and E-cadherin at the cytokine and gene expression levels using ELISA and RT-PCR, respectively. This study involves elucidating the relationship between potential inflammatory signaling molecules and the EMT mechanism as a result of HCF treatment on Caco-2 and CoEpi cell lines.

## Material & Methods

### Preparation of Hydatid Cyst Fluid

HCFs were obtained from animals identified with hydatid cysts during routine slaughters at slaughterhouses by aspiration with a syringe under aseptic conditions. Cyst fluid was examined microscopically for the presence of protoscolices between the slide and coverslip. In addition, DNA isolation from cyst fluid for *E. granulosus* s.l. species confirmation and genotype determination was performed using the Thermo Blood and Tissue DNA Extraction Kit (Qiagen, Hilden, Germany) according to the manufacturer’s instructions. The extracted DNA was subjected to PCR targeting an ~ 875 bp region of the cytochrome oxidase 1 (mt-CO1) gene. Amplicons observed as positive in electrophoresis were analyzed by DNA sequencing, and the results were evaluated using the BLAST algorithm.

### Culture of Cell Lines

Human colorectal adenocarcinoma (Caco-2, HTB-37™ ATCC, U.S.A) cell line and the human colon epithelial (CoEpi, CRL-1459™ ATCC, U.S.A) cell lines were used in the study. The cells were suspended in a DMEM medium containing 10% fetal bovine serum (FBS), supplemented with antibiotics (100 U/ml penicillin, 100 µg/ml streptomycin). Afterward, cells were incubated in an incubator containing 5% CO_2_ to regulate their metabolic activities.

### Treatment of Caco-2 and CoEpi Cell Lines with Hydatid Cyst Fluid

The Caco-2 and CoEpi cell lines seeded in flasks were treated with HCF at dilution doses of 1/2, 1/3, and 1/5 volumes. No HCF treatment was applied to the control groups. The cells were then incubated at 37 °C in an environment containing 5% CO_2_ for 24 h to regulate their metabolic activities.

The widest viability window in a pilot XTT screen was obtained with dilutions of 1/2, 1/3, and 1/5 (v/v), which correspond to the ranges that modulated EMT markers in our earlier Caco-2 work [5]. Because cytokine/EMT gene responses peak between 16 and 30 h before nutrient depletion, a single 24-hour exposure was selected.

### Cell Proliferation Assay (XTT)

The water-soluble tetrazolium XTT assay, which is functionally equivalent to MTT but preserves cells for subsequent RNA and protein analyses, was used to measure cell viability. The cells were seeded in 96-well cell culture plates at approximately 5 × 10^4^ cells/well. After being treated with the specified HCF dilution doses, the Caco-2 and CoEpi cell groups were incubated for 24 h in an incubator containing 5% CO_2_. After incubation, 50 µl of XTT solution was added to each well. Following the addition of the solution, the cells were incubated again at 37 °C for 2 h. After the incubation period, the optical density (OD) of the tested groups was measured at a wavelength of 450 nm using an ELISA plate reader. The results were normalized by comparing them to the control group values.

### Total RNA Extraction

Total RNA extraction from cell application groups was performed using the Hybrid-R™ kit (Lot No: 30521J29036, GeneAll^®^, Korea). Caco-2 and CoEpi cells were seeded in 6-well cell culture flasks at approximately 3 × 10^5^ cells/well. Total RNA was isolated from the cells with the extraction kit according to the manufacturer’s instructions. Firstly, 1 ml RiboEx was added to each well. The collected cells were transferred to an Eppendorf tube. Chloroform was added, mixed using a vortex device, and then incubated. Following the incubation period, a centrifuge was performed at + 4 °C, 12,000 x*g* for 15 min. The upper phase was transferred to a sterile Eppendorf tube. Buffer RBI was then added to the tube and transferred to an F-Type Mini Column. Subsequently, Buffer SWI and Buffer RNW were added in sequence, with centrifugation after each buffer addition. Finally, 60 µl of RNase-free water was added to the top of the column and centrifuged at 10,000 x*g* for 1 min. The isolated RNAs were stored at -20 °C. The purity of RNA in each sample was determined to assess the quality of cDNA synthesis after isolation.

### Real‑Time Polymerase Chain Reaction (RT-PCR)

The cDNA synthesis from the isolated RNA was performed using the Applied biosystems™ (ThermoFisher, Lithuania) cDNA RT kit. For each RNA sample (10 µl) to be used for cDNA synthesis, a single-step Reverse Transcription PCR reaction mixture was prepared by adding all components in the specified amounts on ice. The prepared mixture was distributed to all samples with 10 µl per sample, ensuring that the volume of each reaction was 20 µl After cDNA synthesis, gene expression levels were determined using a 96-well plate with SYBR Green PCR Master Mix on a ViiA7 Real-Time PCR (Applied Biosystems) device for RT-PCR analysis. In the RT-PCR process, the primers used are provided in Table 1 below. In all the experiments conducted, the value of the investigated gene was normalized by calculating the ratio to the control gene (housekeeping gene, GAPDH) values.

**Table 1:** Primers used in RT-PCR

Primer	Sequence
GAPDH	**F**: **5′**-ATGGGCAGCCGTTAGGAAA-3′ **R**: 5′-GCATCGCCCCACTTGATTTT-3′
IL-4	**F**: 5’-CATGCATGGAGCTGCCTGTA-3’ **R**: 5’-AATTCCAACCCTGCAGAAGGT-3’
TNF-α	**F**: 5′-ATCCTGGGGGACCCAATGTA-3′ **R**: 5′-AAAAGAAGGCACAGAGGCCA-3′
TGF-β1	**F**: 5′-TGGTGGAAACCCACAACGAA-3′ **R**: 5′-AGAAGTTGGCATGGTAGCCC-3′
Vimentin	**F**: 5’-CCAAGACACTATTGGCCGCCTGC-3’ **R**::5’-GCAGAGAAATCCTGCTCTCCTCGC-3’
E-Cadherin	**F**: 5’-TGGGCCAGGAAATCACATCCTACA-3’ **R**: 5’-TTGGCAGTGTCTCTCCAAATCCGA-3’

### ELISA Analyzes

For the ELISA test, cells seeded in 6-well flasks at a density of 3 × 10⁵ cells/well in a complete medium were collected according to the kit instructions (Bioassay Technology Laboratory, China). Briefly, 40 µl of cell culture samples were added to the sample wells. Subsequently, 10 µl of antibody was added. Following the addition of the antibody, 50 µl of streptavidin-HRP was added to the sample and standard wells (not added to the standard control well). After pipetting within the plate, the plate cover was closed, and it was incubated at 37 °C for 60 min. After incubation, 300 µl of wash buffer was added to the wells for each wash and left to sit for 30 s. This process was repeated 5 times, washing all wells. Afterward, the plate was dried using an absorbent paper towel. After the washing process, 50 µl of substrate solution A was added to each well, followed by the addition of substrate solution B. Subsequently, the plate was incubated in the dark at 37 °C for 10 min. After incubation, a stop solution was added to each well, and the color change from blue to yellow was observed. Finally, the optical density (OD) value was measured at a wavelength of 450 nm using an ELISA reader (BMG LabTech, Germany).

### Bicinchoninic Acid (BCA) Assay

For the BCA protein assay, the protein test kit (ABP Biosciences, U.S.A) was used. The experiments were performed using a 96-well plate, following the instructions provided in the kit. Subsequently, the absorbance was measured at a wavelength of 562 nm.

### Statistical Analysis

GraphPad Prism 8.4.3 software was used for the statistical analyses. The results were interpreted using the T-test. A p-value of 0.05 or lower was considered statistically significant.

## Results

### Cell Proliferation Assay (XTT)

**Fig. 1:**
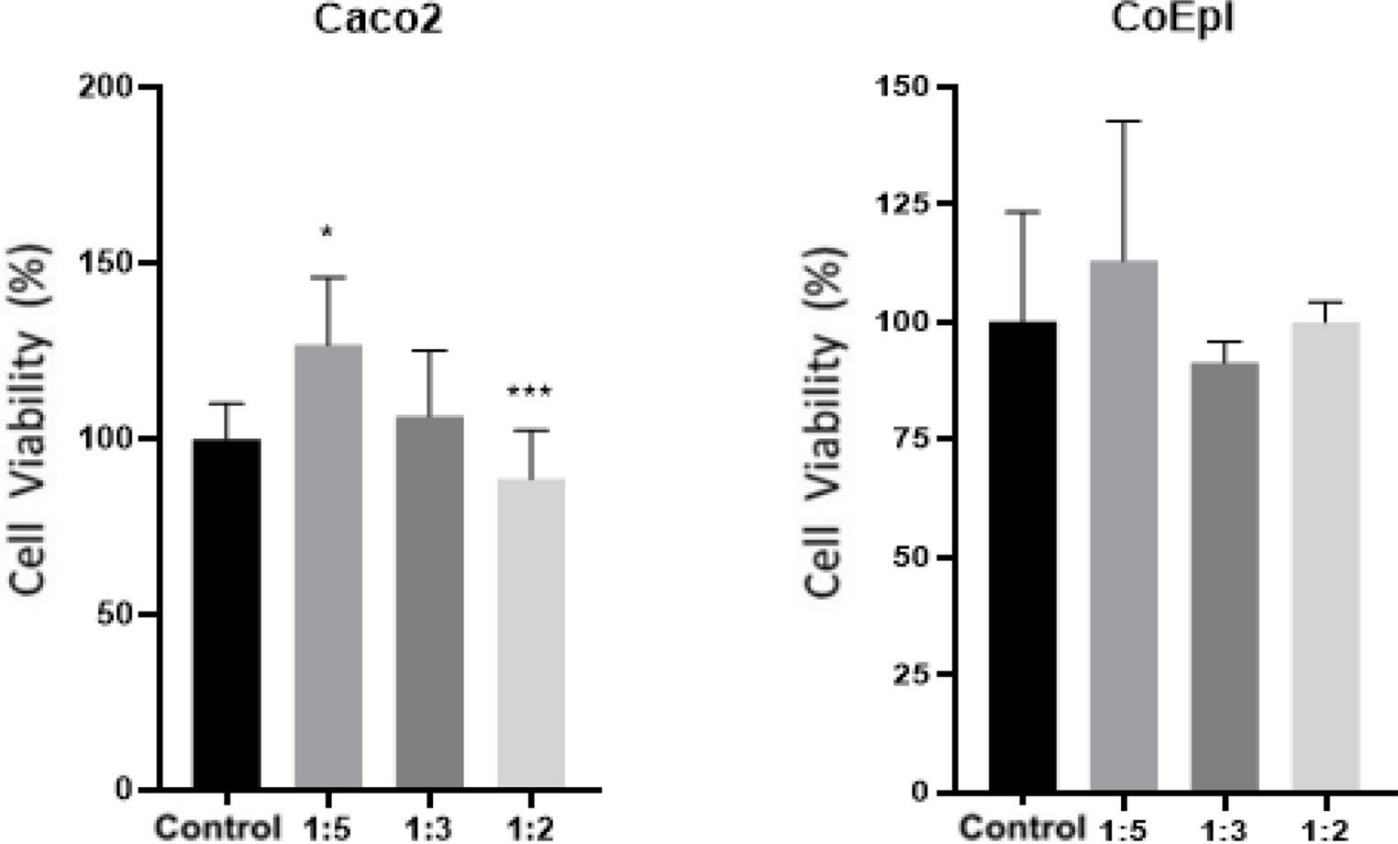
Effect of HCF on cell viability in Caco-2 and CoEpi cells (*P < 0.05, ***P < 0.001)

The effect of HCF treated at various concentrations on the viability of Caco-2 and CoEpi cells was determined by the XTT assay. According to the results of the test, the effect of HCF on cell viability in the Caco-2 cell line, when compared to the control group, showed a significant increase in cell viability in the 1/5 volume treatment group (*P < 0.05). On the other hand, cell viability in the 1/3 treatment group was found to be statistically insignificant (*P* > 0.05). In the 1/2 volume treatment group, a significant decrease in cell viability was observed compared to the control group (***P < 0.001). In the CoEpi cell line, the effect of HCF on cell viability was found to be statistically insignificant in all treatment groups (*P* > 0.05) (See, Fig. 1).

### ELISA

**Fig. 2:**
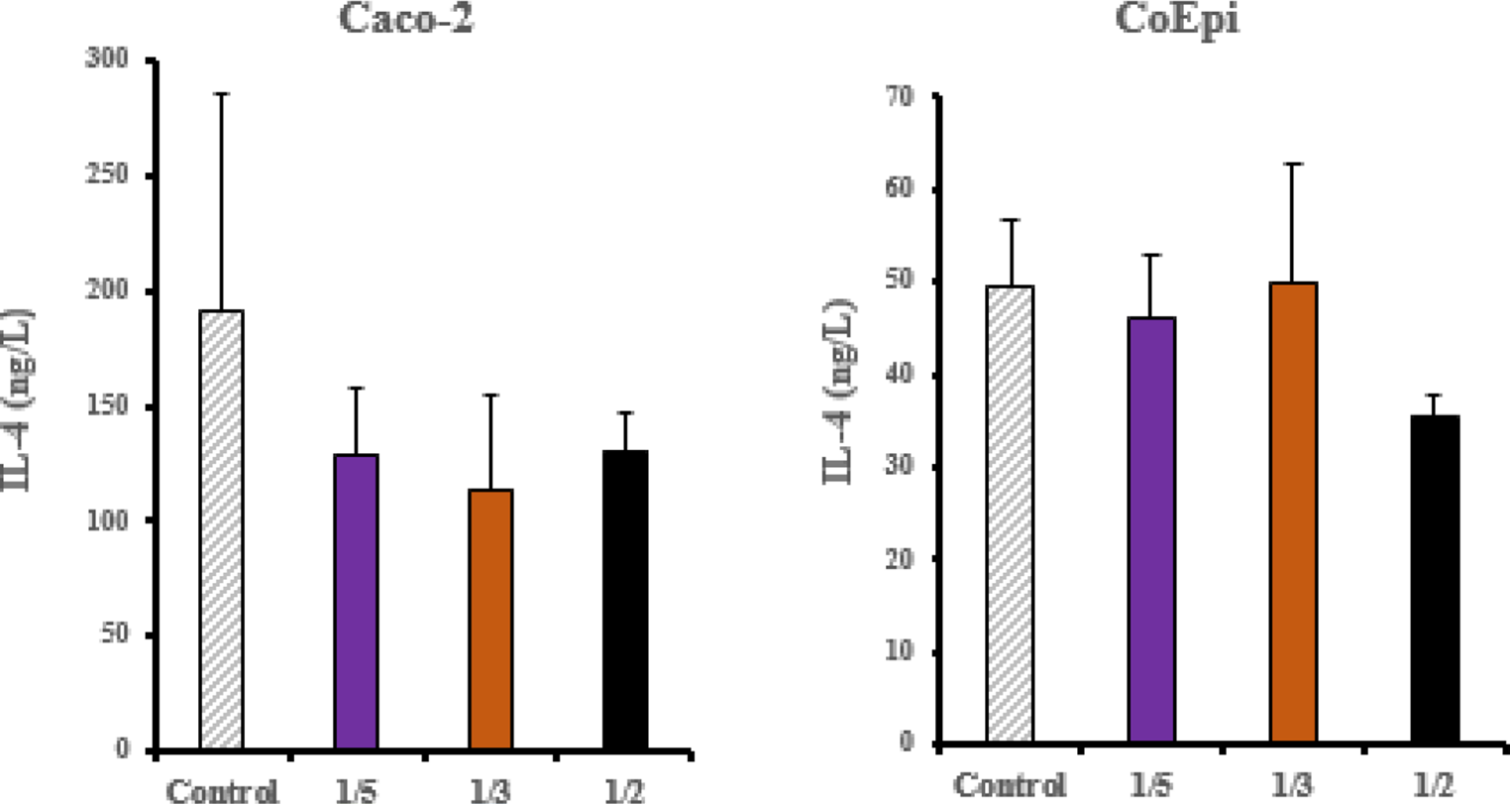
IL-4 ELISA results in Caco-2 and CoEpi cells after HCF treatment. (*P* > 0.05, *n* = 3)

After the ELISA tests, a BCA protein assay was performed to determine the protein amount in the treated cell groups. The amount of each cytokine determined in the ELISA experiment was normalized to the BCA protein level, and the analysis results were obtained. In the control group of the CoEpi cell line, no significant difference was observed in IL-4 concentration between the 1/3 and 1/5 volume treatment groups (*P* > 0.05). In the 1/2 volume treatment group, a significant decrease in cytokine concentration was observed compared to the control (* *P* < 0.05). In the ELISA test for the Caco-2 cells, no statistically significant difference in IL-4 cytokine concentration was observed in any treatment group compared to the control (*P* > 0.05) (Fig. 2).

In the CoEpi cell line, a statistically significant difference in TGF-β1 concentration was detected in the 1/2, 1/3, 1/5 groups compared to the control, showing a marked downward trend correlation independent of the application volume ratio (*** *P* < 0.001, ** *P* < 0.05,). In the Caco-2 cell line, a statistically significant decrease in TGF-β1 cytokine levels was detected in the All application groups compared to the control group (*** *P* < 0.001, ** *P* < 0.05).

**Fig. 3:**
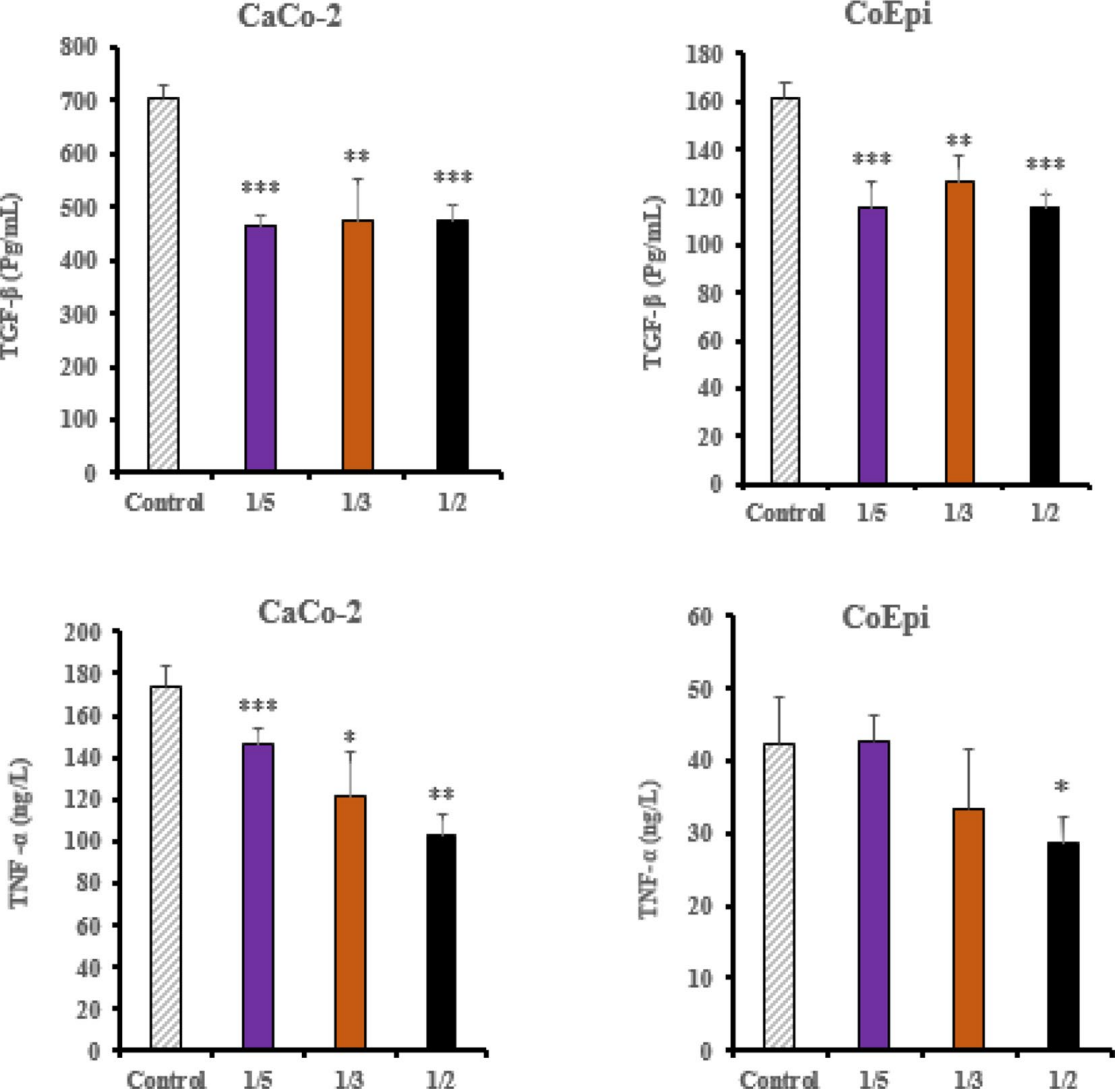
TNF-α and TGF-β ELISA results in Caco-2 and CoEpi cells after HCF treatment (*** *P* < 0.001, ** *P* < 0.01, * *P* < 0.05, *n* = 3)

In the CoEpi cell line, a statistically significant difference in TNF-α concentration was detected in the 1/2 application group compared to the control group (* *P* < 0.05). However, no significant difference was detected in the 1/3 and 1/5 application groups (*P* > 0.05). In the Caco-2 cell line, a statistically significant decrease in TNF-α concentration was observed in the all application groups compared to the control group (*** *P* < 0.001, ** *P* < 0.01, * *P* < 0.05) (Fig. 3).

### RT-PCR

**Fig. 4:**
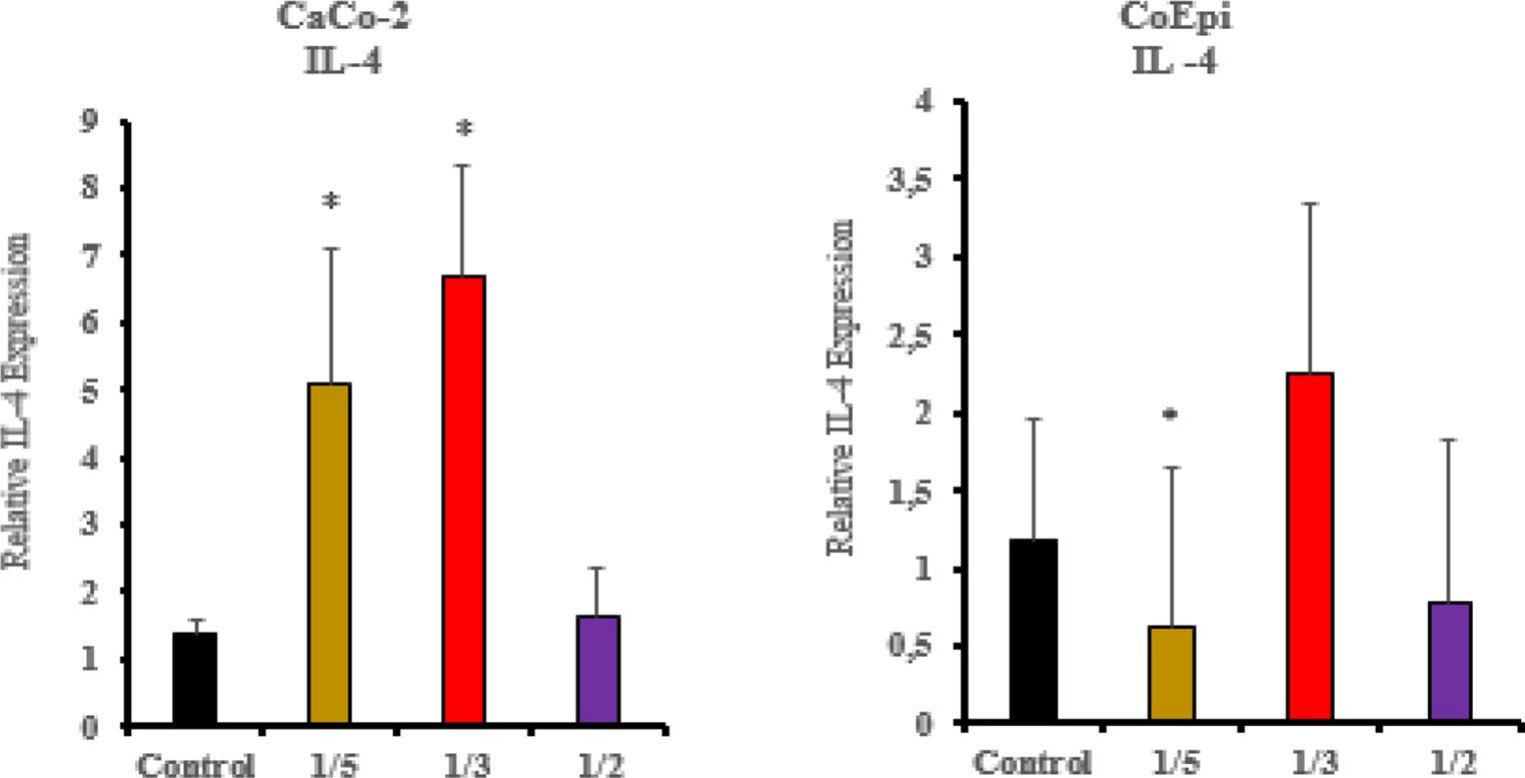
IL-4 gene expression levels after applying HCF to Caco-2 and CoEpi cells (*P < 0.05, *n* = 3)

Gene expression values of the cell groups treated with HCF were determined, and their activities were compared. To investigate the relationship between the inflammatory mechanism and EMT, the primer sequences for IL-4, TNF-α, TGF-β, vimentin, and E-cadherin (Table 1) were used. In the Caco-2 cells, IL-4 gene expression remained the same as the control group at the 1/2 volume concentration, while the gene expression level increased (Fig. 4) in the 1/3 and 1/5 volume HCF treatment groups (* *P* < 0.05). In the CoEpi cells, compared to the control group, a significant decrease in IL-4 gene expression resulted in the 1/5 volume administration group, Independently of concentration volume ratios (* *P* < 0.05).

In the Caco-2 cells, a significant decrease in gene expression levels of TGF-β1 was shown in the 1/2 and 1/3 volume treatment groups of the HCF application compared to the control (* *P* < 0.05, *** *P* < 0.001). At the same time, a significant increase in TGF-β1 gene expression levels was observed in the 1/5 volume treatment group (** *P* < 0.01). In the HCF treatment groups of the CoEpi cell line, a significant downward correlation in gene expression levels was observed in the 1/2, 1/3, and 1/5 volume groups compared to the control group (* *P* < 0.05).

**Fig. 5:**
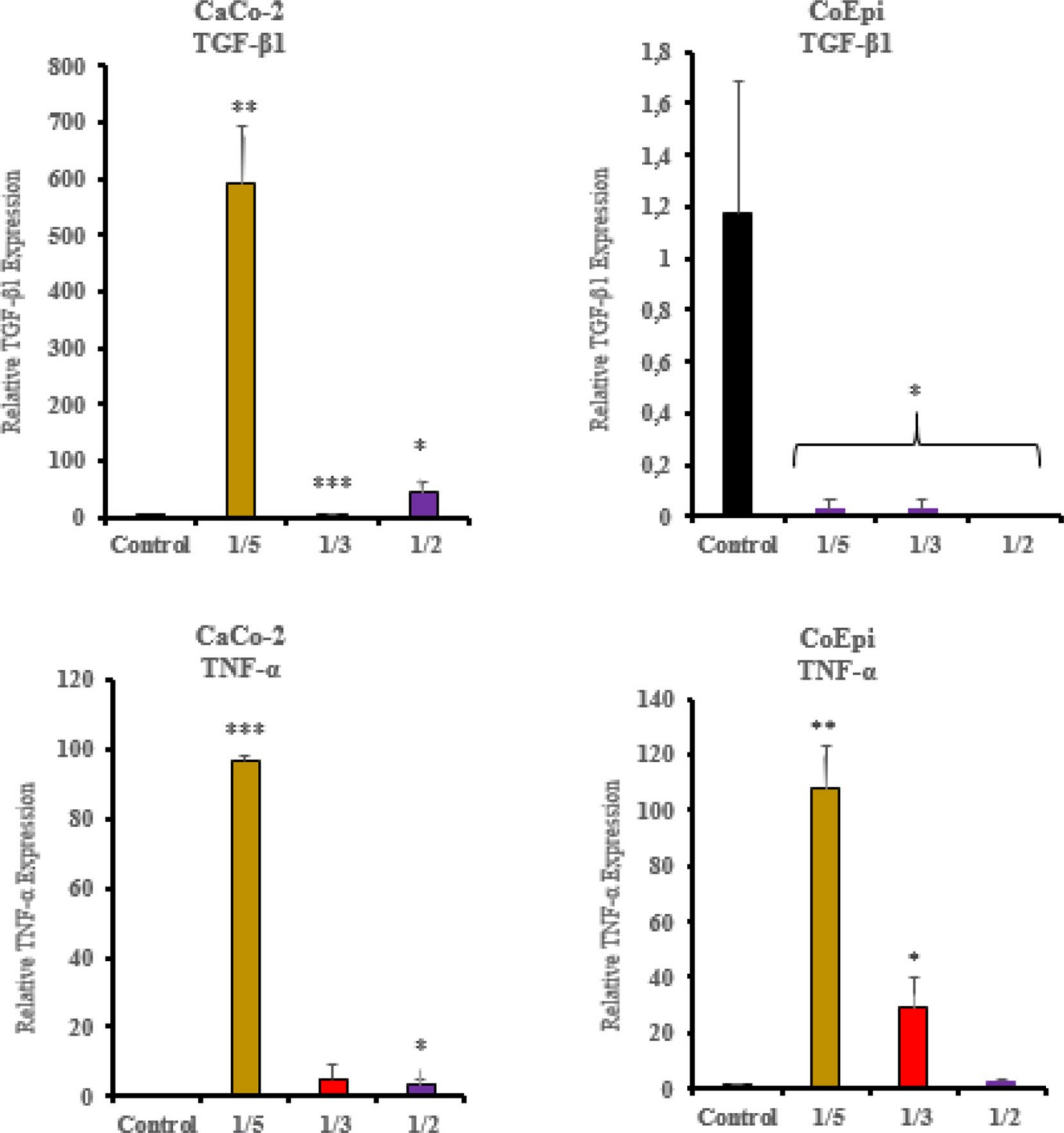
TGF-β1 and TNF-α gene expression levels after the application of HCF to Caco-2 and CoEpi cells (*** *P* < 0.001, ** *P* < 0.01, * *P* < 0.05, *n* = 3)

The TNF-α gene expression level in the Caco-2 cells showed a significant increase in the 1/2 volume treatment group compared to the control group (* *P* < 0.05). However, no significant change in expression level was observed in the 1/3 volume treatment group (*P* > 0.05). On the other hand, a persistent increase in gene expression level was observed in the 1/5 volume treatment group (*** *P* < 0.001). In the CoEpi cells, gene expression levels in the 1/2 volume treatment group were found to be non significant compared to the control group (*P* > 0.05). Conversely, in the 1/3 and 1/5 volume treatment groups, the increase in TNF-α gene expression levels was found to be statistically significant (* *P* < 0.05, ** *P* < 0.01) (Fig. 5).

**Fig. 6:**
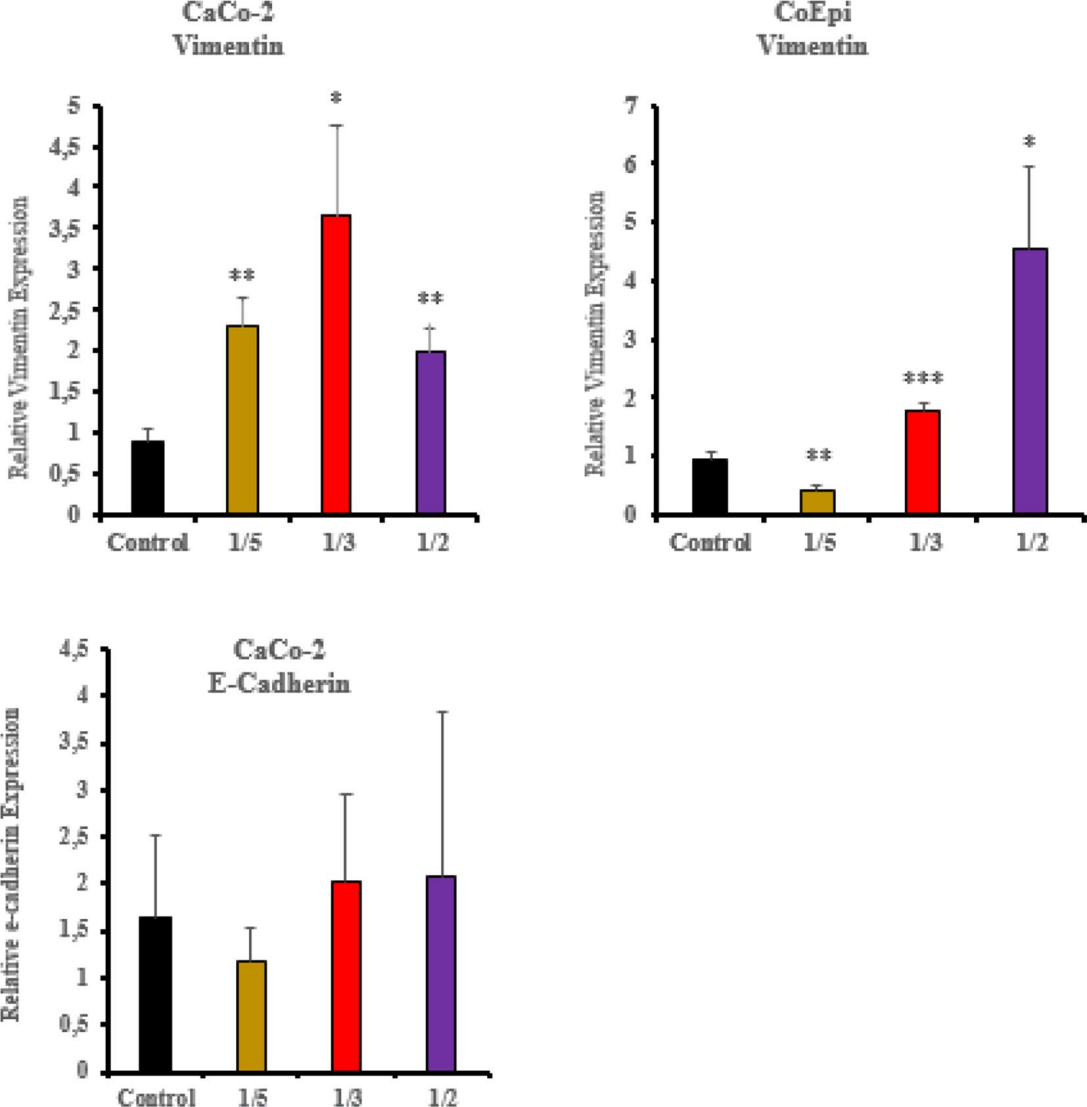
Vimentin and E-Cadherin gene expression levels after the application of HCF to Caco-2 and CoEpi cells (*** *P* < 0.001, ** *P* < 0.01, * *P* < 0.05, *n* = 3)

In the Caco-2 cell treatment groups, the vimentin gene expression levels were higher in the 1/3 and 1/5 volume treatment groups compared to the control group (** *P* < 0.01), and the increase was more pronounced than in the 1/2 volume treatment group (* *P* < 0.05). In the CoEpi cells, the vimentin gene expression levels in the 1/3 and 1/5 volume treatment groups were similar to the control group, while in the 1/2 volume treatment group, the expression level was significantly higher than in the control group (* *P* < 0.05). In the Caco-2 cells, no statistically significant persistent result was obtained for the E-cadherin gene expression levels in the treatment groups (*P* > 0.05). In order to E-Cadherin is used as a marker for EMT transition in cancer cells, its expression levels in healthy colon epithelial cells were not included (Fig. 6).

## Discussion

It has been reported that some parasitic infections suppress certain types of tumor formation, as indicated by various studies [11]. In addition, recent study data have shown that in infections caused by *E. granulosus*, randomly diagnosed hydatid cyst cases are lower among solid tumor cancer patients compared to other cases [12]. On the other hand, the relationship between *Echinococcus* and cancer remains uncertain, with some contradictory results. According to current data, studies conducted by various groups have documented in the literature that hydatid cyst protoscoleces have a direct or indirect anti-cancer effect within the tumor microenvironment [13]. For example, protoscoleces have exhibited anti-cancer activity by inducing cell death in WEHI-164 fibrosarcoma cells [14]. According to the results of another study, although the application of HCF did not directly inhibit cell proliferation in the human lung adenocarcinoma (A549) cell line, it was appointed that the A549 cells became more sensitive to apoptosis [15]. In another study conducted with HCF treated Caco-2 cell line, an increase in cell proliferation was observed along with a decrease in p53 expression. Furthermore, a reduction in apoptosis was found [5]. This study aimed to investigate the anti-tumorigenic or pro-tumorigenic effects of HCF at various concentrations on Caco-2 and CoEpi cell lines concerning inflammatory mediators at the molecular level. The relationship between HCF application and EMT transition in the Caco-2 cell line has been emphasized in various studies [5, 16]. Since EMT progression imparts a migratory and invasive character to epithelial cells, it significantly contributes to metastasis. Tumor cells with an invasive character are associated with poor prognosis. EMT also involves a mechanism that can be regulated by various anti-inflammatory and pro-inflammatory cytokines [17]. TGF-β1, vimentin and E-cadherin signaling proteins are reported to be the best-known regulators of EMT progression [18, 19]. Anti-cancer signals directly triggered by parasite antigens activate host defense mechanisms, leading to the initiation of various immunological processes. Many reports have discussed the common relationship between parasites and tumor antigens [18–20]. Caco-2 cell lines are commonly used in in vitro EMT studies due to their ability to differentiate into mature intestinal cells [21]. Additionally, it holds the distinction of being the first of its kind in the literature. Within the scope of the study, it is planned to investigate the potential cytotoxic or carcinogenic effects of HCF on cell lines at the molecular level through comparison. On the other hand, investigating the inflammatory mechanism could be an important step for cancer treatments. The molecular study of parasite antigens from *E. granulosus* and the antigen-receptor relationship in Caco-2 cells could be used as an immunotherapeutic tool to control the metastasis and viability of malignant tumor cells.

To simulate real-world exposure, we used unfractionated HCF; single fractions frequently lose activity, suggesting a synergy between components derived from the parasite and the host [8, 9]. In the study, the comparison and analysis of different HCF application volumes were performed. After HCF application in CoEpi cells, no significant difference was observed in the E-cadherin gene expression levels compared to the control group. On the other hand, when evaluating data in Caco-2 cells, a decrease in E-cadherin levels was observed in the 1/5 volume application group, while an increase was recorded in the 1/2 volume application group compared to the control. Compared to E-cadherin, the expression profile of the vimentin molecule in mesenchymal cells after transition to the EMT model is important for determining mesenchymal cell characteristics. In the Caco-2 cells, vimentin gene expression levels increased in the 1/5 and 1/3 application groups, regardless of volume ratio, compared to the control and the 1/2 concentration volume groups. (*P* < 0.01). Compared to CoEpi cells, the decrease in vimentin in the 1/5 volume application group was found to be significant in Caco-2 cells. (*P* < 0.01). According to a recent study report using the Caco-2 cell line in a mesenchymal cell model, a significant increase in vimentin gene expression level was recorded on day 30 when EMT was induced experimentally, with day 0 and day 30 as reference points [22].

In this study, consistent with the vimentin and E-cadherin gene expression results, the XTT cell proliferation assay in the Caco-2 cell line showed a significant increase in cell viability in the 1/5 volume application group (*P* < 0.05). EMT transition has been demonstrated in the 1/5 volume application group following HCF application in the Caco-2 cell line. In this context, pro-inflammatory pathways are activated through the release of certain intracellular cytokines during the enhancement of cancer cell proliferation and metastatic potential. When examining the relationship between TGF-β1, E-cadherin and vimentin, which are important markers for EMT, a significant decrease in TGF-β1 cytokine levels in the ELISA test was found in the 1/2 volume HCF application group in Caco-2 cells compared to CoEpi cells (*P* < 0.001). Supporting the ELISA test results, a significant decrease in TGF-β1 gene expression levels was also observed in the 1/2 volume application group (*P* < 0.05). On the other hand, in the Caco-2 cell line, an increase in TGF-β1 gene expression levels was observed in the 1/5 volume application group, independent of the ELISA test results (*P* < 0.01). Following the results from the XTT cell viability test, cell viability significantly decreased in the 1/2 volume application group (*P* < 0.001), while a significant increase in cell viability was observed in the 1/5 volume application group (*P* < 0.05). Many studies have investigated the role of TGF-β1 signaling molecules in EMT in in vitro models. It has been reported that, in the intermediate and late stages, TGF-β1 has an inductive effect on EMT in cancer cells and in this context, it acts as a pro-inflammatory factor, contributing to cancer progression and an increase in invasive potential [23]. In this case, as a result of the treatment of cell groups with HCF, TGF-β1 demonstrated a pro-inflammatory effect on Caco-2 cells compared to the CoEpi cell line. It was explained that this effect, in connection with vimentin and E-cadherin gene expression levels and the cell proliferation test, supported EMT transition in the 1/5 volume application group [24, 25].

Various reports provide significant evidence that TNF-α / TGF-β1 also regulates several receptor elements of the signaling mechanism in the tumor microenvironment in an interrelated manner. After HCF application in the Caco-2 cell line, a decrease in TNF-α was recorded in the 1/2 volume application group compared to the CoEpi cell line (*P* < 0.05). In the ELISA, TNF-α cytokine test, the decrease in the 1/2 volume application group was found to be significant compared to the control group (*P* < 0.001). Consistent with the XTT test, TNF-α levels showed a decrease in both the ELISA and RT-PCR results in the 1/2 volume application group. On the other hand, in the 1/5 volume HCF application group, the gene expression level in Caco-2 cells was found to be higher compared to the control group (*P* < 0.001). TNF-α increases the transcription of various inflammatory mediators and inflammation-supporting intracellular downstream molecules in most malignant tumors, thereby providing tumor cells with an invasive character and it has been shown to promote the progression of the metastatic process in Caco-2 cells [26]. According to data from previous studies, TNF-α and TGF-β1 can stimulate each other’s production. Additionally, TNF-α has been shown to affect TGF-β1 expression in many cells and tissues. In our study, TNF-α and TGF-β1 gene expression levels and cytokine levels are correlated. In this context, research supports that TGF-β1 and TNF-α can initiate each other’s activation in in vitro experiments [27]. No significant relationship was found between IL-4, an anti-inflammatory cytokine and cell viability. In colorectal cancer, IL-4 plays context-dependent roles in both tumor promotion and tumor suppression. It also controls the Th2 response to helminth antigens [9]. Additionally, proteins and RNA have different clocks. Because IL-4/STAT6 signaling triggers the destabilizing factor tristetraprolin (TTP), IL-4 mRNA bursts early (≤ 4 h) and is quickly degraded [28]. When transcripts have already diminished, ELISA can still detect secreted IL-4 protein because it is much more stable and accumulates in the medium (half-life ≥ 6 h). These mRNA vs. protein mismatches are typical for cytokines, according to large vaccine datasets [29]. Further analysis of the gene and protein levels is required to comprehensively understand the various components of the intracellular inflammatory mechanism. Additionally, there is a need for more comprehensive molecular-level analyses by evaluating the shared antigen-receptor structure between cancer and *E. granulosus* using several different analytical methods. Future research is planned to refine the antiproliferative read-out using flow-cytometric annexin-V and cell-cycle assays.

In conclusion, following HCF application in Caco-2 cell line, EMT transition was successfully detected in the 1/5 concentration volume application group, as indicated by ELISA, qRT-PCR and cell proliferation test results. On the other hand, a linear relationship was demonstrated between decreases in TGF-β1 and TNF-α levels, which regulate the pro-inflammatory signaling mechanism based on the cell microenvironment, and reduced cell viability. As the HCF concentration decreases (1/5), cell viability increases, while TGF-β1 and TNF-α levels show an increase. In this case, low-concentration HCF promotes cancer activity by inducing EMT transition in Caco-2 cells. As the HCF application concentrations increase (1/2) in Caco-2 cells, a significant decrease in TGF-β and TNF-α levels as well as cell viability, has been observed. It can be noted that TGF-β1 and TNF-α may enhance the metastatic phenotype in Caco-2 cells and initiate the release of inflammatory mediators. For this reason, high-concentration HCF exhibits an anti-cancer effect on Caco-2 cells. On the other hand, we also consider that it may have a protagonistic effect on cell proliferation. In this context, the mutual antigen-receptor structure between *E. granulosus* and cancer may modulate the signals of the immune response it regulates for tumor cell progression by affecting immune system cells. The antigen-receptor structure can be considered an immunological tool, as it may provide cross-protection by halting cancer cell proliferation and inducing apoptosis. After HCF application, the parasitic antigens of *E. granulosus* may represent a fundamental aspect of our study to gain a deeper understanding of the anti-cancer activity of Caco-2 cells and the inflammatory mediators involved.

## Data Availability

No datasets were generated or analysed during the current study.
